# Postnatal Serotonin Type 2 Receptor Blockade Prevents the Emergence of Anxiety Behavior, Dysregulated Stress-Induced Immediate Early Gene Responses, and Specific Transcriptional Changes that Arise Following Early Life Stress

**DOI:** 10.1016/j.biopsych.2011.08.005

**Published:** 2011-12-01

**Authors:** Madhurima Benekareddy, Krishna C. Vadodaria, Amrita R. Nair, Vidita A. Vaidya

**Affiliations:** Department of Biological Sciences, Tata Institute of Fundamental Research, Mumbai, India

**Keywords:** Activity-regulated cytoskeletal-associated protein (*Arc*), habituation, immobilization stress, ketanserin, maternal separation, prefrontal cortex

## Abstract

**Background:**

Early life adverse experience contributes to an enhanced vulnerability for adult psychopathology. Recent evidence indicates that serotonin type 2 (5-HT_2_) receptor function, implicated in the pathophysiology of mood and anxiety disorders, is significantly enhanced in the maternal separation model of early life stress. We examined whether postnatal 5-HT_2_ receptor blockade would prevent the consequences of maternal separation on anxiety behavior and dysregulated gene expression.

**Methods:**

Control and maternally separated litters received treatment with the 5-HT_2_ receptor antagonist, ketanserin, or vehicle during postnatal life and were examined for effects on adult anxiety behavior, adult stress-induced immediate early gene expression responses, and transcriptional changes within the prefrontal cortex during postnatal life and in adulthood.

**Results:**

Treatment with ketanserin during postnatal life blocked the long-lasting effects of maternal separation on anxiety behavior in the open field test and the elevated plus maze. Further, the dysregulated adult stress-induced expression pattern of the immediate early gene, *Arc*, observed in maternally separated animals was also prevented by postnatal ketanserin treatment. Ketanserin treatment normalized the alterations in the expression of specific genes in the prefrontal cortex of maternally separated animals, including changes in serotonin type 2A receptor messenger RNA expression during postnatal life and in genes associated with G-protein signaling in adulthood.

**Conclusions:**

Postnatal treatment with the 5-HT_2_ receptor antagonist, ketanserin, blocked specific consequences of maternal separation, including anxiety behavior and dysregulated gene expression in the prefrontal cortex. Our results suggest that enhanced 5-HT_2_ receptor function may contribute to the emergence of anxiety behavior and perturbed stress responses following early life stress.

A history of early life adverse experience is a major risk factor for the development of anxiety and depressive disorders (1–3). Rodent models of early life stress, such as maternal separation (MS), exhibit endophenotypes of these affective disorders (4–6). MS, which involves daily 3-hour long separations of the litter from their dam from postnatal days 2 to 14, results in enhanced adult anxiety behavior (7–9). Further, MS animals exhibit perturbed neuroendocrine stress responses in adulthood (7,10,11), which could serve to disrupt homeostasis and exacerbate the risk for psychopathology. The consequences of MS have been reported to endure across the life span, indicating the relatively persistent nature of the changes in emotionality.

Serotonergic neurocircuitry is known to be involved in the development of anxiety (12–14). Serotonin type 2 (5-HT_2_) receptors have been implicated as possible targets to modulate anxiety behavior. Serotonin type 2A (5-HT_2A_) and serotonin type 2C (5-HT_2C_) receptor knockouts are reported to exhibit reduced anxiety responses, suggesting a role for the 5-HT_2_ receptors in establishing a baseline anxiety state (15,16). However, it remains unknown whether 5-HT_2_ receptors contribute to the enhanced anxiety and perturbed stress responses that arise following early life stress exposure. Recent evidence indicates that MS animals exhibit significantly enhanced 5-HT_2_ receptor function in the prefrontal cortex (PFC) and increased 5-HT_2_ receptor driven behavioral responses (17). We hypothesized that pharmacological blockade of the 5-HT_2_ receptor during MS may prevent several of the long-term sequelae of this early life stress exposure, including the development of enhanced anxiety. Here, we show that postnatal 5-HT_2_ receptor blockade prevents the emergence of specific consequences of MS, namely enhanced anxiety behavior observed in adulthood, dysregulated immediate early gene (IEG) responses to adult-onset stress, and specific transcriptional changes in the PFC of MS animals in both postnatal life and adulthood. Our results implicate the 5-HT_2_ receptor in the development of altered emotionality that arises following early life adverse experience.

## Methods and Materials

### Animal Treatment Paradigms

Sprague-Dawley rats bred in the Tata Institute of Fundamental Research animal facility were group housed and maintained on a 12-hour light-dark cycle with access to food and water ad libitum. Animal procedures were carried out in accordance with the National Institutes of Health Guide for the Care and Use of Laboratory Animals and approved by the Tata Institute of Fundamental Research Institutional Animal Ethics Committee.

Pregnant primiparous female rats delivered litters and were assigned randomly to control or MS groups. MS involved separation of the pups from the dam for 3 hours daily from postnatal days (P) 2 to 14, as described previously (18). For experiments involving postnatal administration of the 5-HT_2_ receptor antagonist, ketanserin tartrate (5 mg/kg; Sigma, Taufkirchen, Germany), control and MS litters were orally administered vehicle (5% glucose) or ketanserin using a feeding needle before the onset of each separation from P2 to P14. The dose of ketanserin selected was based on prior literature (19,20).

### Behavioral Experiments: Open Field and Elevated Plus Maze

To address the consequences of ketanserin treatment during MS on adult anxiety behavior in the open field (21) and elevated plus maze (22), the experimental design had four treatment groups: control with vehicle, control with ketanserin (Ket), MS with vehicle (MS), and MS with ketanserin (MS + Ket) (*n* = 4–7 per group). Animals were placed in the open field (100 cm × 100 cm × 70 cm) in one corner facing the center and allowed to explore the arena for 15 minutes. The elevated plus maze had a height of 50 cm with two open and two closed arms (50 × 10 cm). Animals were placed in the center of the maze, facing an open arm, and allowed to explore for 30 minutes. The behavior was recorded using a charge-coupled device camera and analyzed using the automated Ethovision tracking system (Noldus, Wageningen, The Netherlands).

### Adult-Onset Stress Experiments

To examine whether MS altered the pattern of IEG responses to adult-onset stress, control and MS animals in adulthood (P90) received acute immobilization stress (AIS) (2 hours) or chronic immobilization stress (CIS) (2 hours daily for 10 days). Immobilization stress involved placing the animals in rodent restrainer cones (Stoelting Company, Wood Dale, Illinois). Treatment groups were as follows: 1) AIS experiment: control, AIS, MS, MS + AIS (*n* = 3–5 per group); and 2) CIS experiment: control, CIS, MS, MS + CIS (*n* = 3–5 per group). We next addressed whether ketanserin treatment during MS would normalize the perturbed IEG response in MS animals subjected to CIS. Treatment groups were as follows: vehicle-treated cohort: control + vehicle, MS + vehicle, control + vehicle + CIS, MS + vehicle + CIS; and ketanserin-treated cohort: control + ketanserin, MS + ketanserin, control + ketanserin + CIS, MS + ketanserin + CIS (*n* = 5–10 per group). Animals were sacrificed by rapid decapitation and brains were dissected and stored at −70°C until further processing.

### Gene Expression Analysis: In Situ Hybridization and Quantitative Polymerase Chain Reaction

In situ hybridization was performed to examine expression of the IEG and activity-regulated cytoskeletal-associated protein (*Arc*) in the PFC following AIS and CIS in control and MS animals and to examine whether ketanserin treatment altered the adult stress-induced IEG response (18). In situ hybridization was also carried out to assess 5-HT_2A_ and 5-HT_2C_ receptor expression in the PFC at P10 and P14 in control, MS, and MS + Ket groups (*n* = 3–6 per group). In brief, cryostat-cut sections (14 μm) were thaw-mounted onto ribonuclease free Probe-on plus slides (Electron Microscopy Services, Columbia, Maryland) before fixation. ^35^S-UTP labeled (Amersham, Buckinghamshire, United Kingdom) riboprobes against *Arc* were generated from a transcription-competent plasmid kindly provided by Dr. O. Steward (Johns Hopkins University) and against 5-HT_2A_ and 5-HT_2C_ receptors from templates generated by polymerase chain reaction (PCR) amplification from rat complementary DNA with specific primers containing T3 and T7 template sequences (5-HT_2A_ forward: AATTAACCCTCACTAAAGGGCTGGTCATCATGGCAGTGTC; reverse: TAATACGACTCACTATAGGGTCTGAGGGAGGAAGCTGAAG; 5-HT_2C_ forward AATTAACCCTCACTAAAGGGTAATCGGCCTATTGGTTTGG; reverse: TAATACGACTCACTATAGGGTCACGAACACTTTGCTTTCG). Sections were incubated with the ^35^S-UTP-labeled riboprobe (1 × 10^6^cpm/slide) for 20 hours at 60°C, followed by ribonuclease A (RNase A, 20 μg/mL; USB Corporation, Cleveland, Ohio) treatment and stringent washes. Slides were dried and exposed to Hyperfilm β-max (Amersham) for 5 days. Messenger RNA (mRNA) levels were quantitated using the Macintosh-based Scion Image Software (Scion, Frederick, Maryland) and ^14^C standards for calibration. For *Arc* mRNA levels, equivalent areas of the PFC were outlined for optical density measurements. For 5-HT_2A_ and 5-HT_2C_ mRNA expression within the PFC, levels were ascertained in the infralimbic (IL), prelimbic (PL), and cingulate cortex (CC). Optical density values obtained from both sides of 3 to 4 sections for each animal (6–8 measurements) were averaged to obtain a mean value.

To address whether postnatal ketanserin treatment during MS altered gene expression of specific genes (17) in the PFC (*Prkcb1, Ppp3ca, Plek, Plcd4, Nlgn1, Grin2d)* in adulthood, we performed quantitative polymerase chain reaction on animals belonging to the following groups: control with vehicle, MS, Ket, MS + Ket (*n* = 5–10 per group). RNA was extracted using the TRI reagent (Sigma) and reverse transcribed followed by quantitative polymerase chain reaction with Taqman probes (Applied Biosystems, Carlsbad, California). The data were normalized to the average of four housekeeping genes (*18s rRNA, Actb, Gapdh,* and *Hprt*) and changes in the mRNA levels were quantified using the ΔΔCt method (23,24). See Table S1 in Supplement 1 for a list of primers used.

### Statistical Analysis

Statistical analysis was performed using the software Prism (Graphpad, La Jolla, California). Experiments with four groups were subjected to a two-way analysis of variance (ANOVA), followed by a Bonferroni post hoc test. Experiments with three groups were subjected to a one-way ANOVA, followed by a Bonferroni post hoc test. Data that showed a nonparametric distribution were subjected to the Kruskal-Wallis test followed by a Dunn's post hoc test. Significance was determined at *p <* .05.

## Results

### Postnatal Ketanserin Treatment Blocks the Emergence of Adult Anxiety Behavior in Maternally Separated Animals

We have previously shown that MS is associated with significantly enhanced 5-HT_2_ receptor function (17). Here, we sought to address whether postnatal treatment with the 5-HT_2_ receptor antagonist ketanserin prevents the anxiogenic effects of MS in adulthood observed in the open field and elevated plus maze tests.

**Figure 1:**
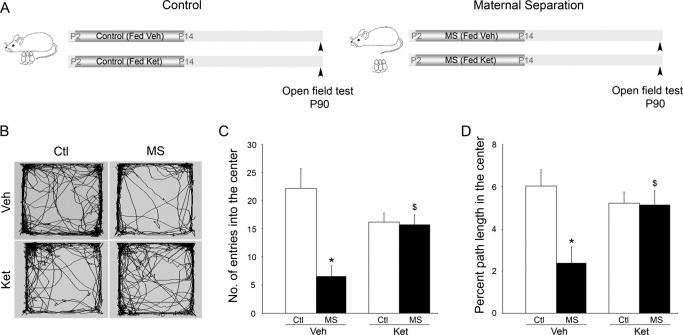
Postnatal treatment with the serotonin type 2 receptor antagonist, ketanserin (Ket), blocks the enhanced adult anxiety responses associated with maternal separation in the open field test. Shown is a schematic for the treatment paradigms **(A)**. Pups from control (Ctl) and maternal separation (MS) groups were fed either vehicle (Veh) or Ket daily from postnatal days (P) 2 to 14 and their behavior was assessed in the open field test in adulthood (P90). **(B)** Shown are representative tracks of the behavior of adult Ctl and MS animals (fed either Veh or Ket) in an open field arena. Maternal separation resulted in a significant decrease in both number of entries **(C)** and the percent path length **(D)** traversed in the center of the open field arena. Postnatal serotonin type 2 receptor blockade with Ket prevented the enhanced anxiety observed in MS animals, as measured by number of entries **(C)** and percent path length **(D)** in the center of the open field. Postnatal Ket treatment to control animals did not alter anxiety behavior as compared with Veh-administered Ctl animals. The results are expressed as the mean ± SEM number of entries into or percent path length in the center of the open field (*n* = 4–7 per group). (*p < .05 as compared with Ctl, ^$^*p* < .05 as compared with MS; analysis of variance and Bonferroni post hoc test).

Postnatal ketanserin treatment blocked the enhanced anxiety responses observed with MS in the open field (Figure 1A–D). Two-way ANOVA analysis revealed a significant MS × Ket interaction for the number of entries [*F*(1,19) = 9.27, *p* = .007] and the percent path length in the center of the open field [*F*(1,19) = 5.48, *p* = .03]. While MS animals exhibited increased anxiety behavior with a significant decrease in both number of entries (Figure 1C) and percent path length (Figure 1D) in the center of the open field, the MS + Ket group showed a blockade of these anxiety responses and were comparable with control animals. The total distance traversed in the open field arena was similar across all treatment groups (Figure S1A in Supplement 1).

**Figure 2:**
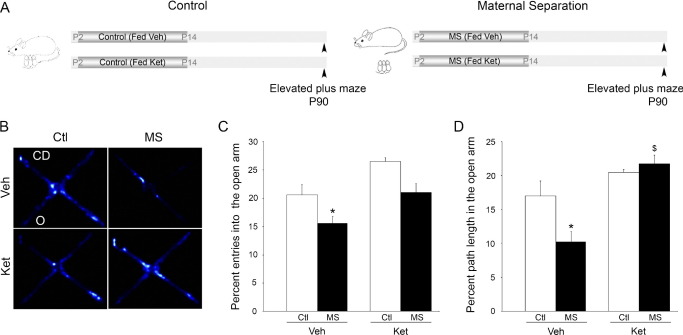
Postnatal treatment with the serotonin type 2 receptor antagonist blocks the increased anxiety behavior observed in maternally separated animals in the elevated plus maze. Shown is the experimental paradigm **(A)** involving postnatal treatment with the serotonin type 2 receptor antagonist ketanserin (Ket) or vehicle (Veh) to rat pups from control (Ctl) or maternal separation (MS) groups. **(B)** Shown are representative heat maps of the time spent in the open (O) and closed arms (CD) of the elevated plus maze. Analysis of the percent entries into the open arms **(C)** and the percent path length in the open arm **(D)** revealed a significant decrease in the MS group. Postnatal treatment with Ket blocked the anxiety behavior following MS, as measured by percent path length **(D)** in the open arms of the elevated plus maze. Postnatal Ket treatment to control animals did not alter anxiety behavior as compared with vehicle-administered Ctl animals. The results are expressed as the mean ± SEM percent entries into or percent path length in the open arm of the elevated plus maze (*n* = 4–7 per group). (*p < .05 compared with control, ^$^*p* < .05 compared with MS; analysis of variance and Bonferroni post hoc test). P, postnatal day.

Postnatal ketanserin treatment also blocked specific anxiety responses of MS animals in the elevated plus maze (Figure 2A–D). Two-way ANOVA analysis revealed a significant MS × Ket interaction in the percent path length traversed in the open arms [*F*(1,19) = 5.21, *p* = .03]. MS animals showed significant increases in anxiety behavior with a decrease in percent path length in the open arms of the maze (Figure 2B, D). This measure of anxiety was blocked in MS animals that received postnatal ketanserin treatment and was comparable with the control group. MS animals also showed a significant decrease in percent entries into the open arms (Figure 2B, C). However, for this measure, two-way ANOVA analysis did not reveal a significant MS × Ket interaction. The total distance traveled in the elevated plus maze was similar across all treatment groups (Figure S1B in Supplement 1). Ketanserin treatment in control animals did not alter baseline anxiety behavior on either the open field or the elevated plus maze.

### Maternal Separation Leads to a Dysregulated Expression of the Immediate Early Gene, *Arc*, in Response to Adult Immobilization Stress

Exposure to diverse stressors, including immobilization stress, is reported to evoke enhanced expression of the 5-HT_2_ receptor regulated IEG, *Arc*, in the PFC (25,26). Given the perturbed prefrontal 5-HT_2_ receptor function in MS animals, we hypothesized that stress-evoked patterns of *Arc* mRNA expression may be aberrant in these animals.

**Figure 3:**
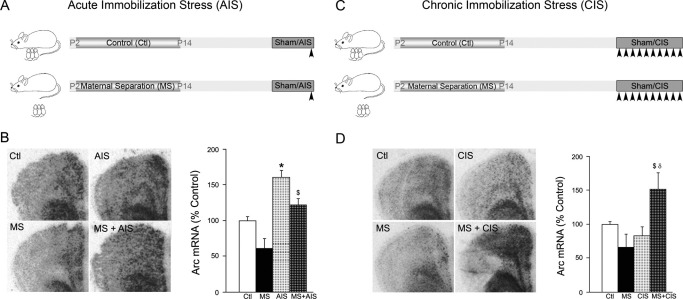
A life history of maternal separation alters the pattern of regulation of the immediate early gene *Arc* within the prefrontal cortex in response to adult-onset chronic immobilization stress. Shown is a schematic for the treatment paradigms **(A, C)**. Pups from Ctl and MS groups were subjected in adulthood to either **(A)** sham or AIS or **(C)** sham or CIS as described in Methods and Materials. In situ hybridization and quantitative densitometric analysis was utilized to assess the levels of *Arc* messenger RNA (mRNA) in the prefrontal cortex. Shown are representative autoradiograms of *Arc* mRNA levels in the prefrontal cortex from Ctl, MS, AIS, and MS + AIS groups **(B)** and from Ctl, MS, CIS, and MS + CIS groups **(D)**. Exposure to AIS in adulthood evoked a similar pattern of *Arc* mRNA regulation in both Ctl and MS animals in the prefrontal cortex **(B)**. Ctl animals subjected to CIS in adulthood showed a habituation of the *Arc* mRNA regulation by stress **(D)**. In striking contrast, MS animals showed a significant induction of prefrontal *Arc* mRNA levels following adult-onset CIS exposure **(D)**. Results are expressed as percent of control and are the mean ± SEM (*n* = 3–5 per group). (*p < .05 compared with Ctl, ^$^*p* < .01 significantly different from MS, ^δ^*p* < .05 significantly different from CIS; analysis of variance and Bonferroni post hoc test). Abbreviations as in Figures 1 and 2.

Acute immobilization stress exposure resulted in a robust induction of prefrontal *Arc* mRNA in both control and MS animals (Figure 3A, B). Two-way ANOVA analysis revealed no significant MS × AIS interaction. MS did not significantly regulate baseline *Arc* mRNA expression in the PFC. This indicates that the AIS-induced pattern of *Arc* mRNA regulation in the PFC does not differ between animals with a normal or adverse early life history. Repeated exposure to a homotypic stressor, including immobilization stress, has been reported to result in the habituation of cortical *Arc* mRNA induction (27). We asked whether MS animals exhibit a similar pattern of habituation in prefrontal *Arc* mRNA induction in response to CIS in adulthood (Figure 3C). Strikingly, MS animals failed to show a habituation of *Arc* mRNA upregulation in the PFC despite repeated exposure to immobilization stress once daily for 10 days in contrast to control animals (Figure 3D). Two-way ANOVA analysis revealed a significant MS × CIS interaction for *Arc* mRNA regulation in the PFC [*F*(1,14) = 15.02; *p* = .0017]. These results demonstrate that a history of MS results in a dysregulated IEG pattern in the PFC following chronic, but not acute, exposure to immobilization stress.

### Postnatal Ketanserin Treatment Normalizes the Dysregulated Pattern of *Arc* mRNA Regulation Observed Following Chronic Stress in Maternally Separated Animals

**Figure 4:**
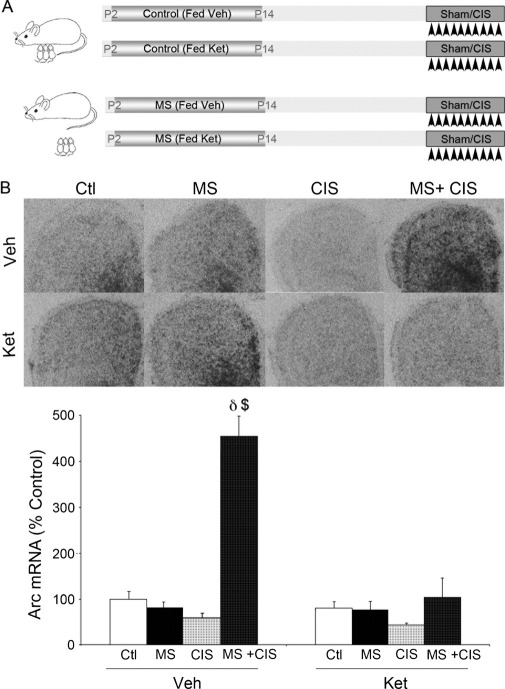
Postnatal serotonin type 2 receptor blockade prevents the dysregulated pattern of expression of the immediate early gene *Arc* within the prefrontal cortex in response to adult-onset chronic immobilization stress observed in maternally separated animals. Shown is the experimental paradigm **(A)** involving postnatal treatment with the serotonin type 2 receptor antagonist ketanserin (Ket) or vehicle (Veh) to rat pups from control (Ctl) or maternal separation (MS) groups followed by exposure in adulthood to sham or chronic immobilization stress (CIS) as described in Methods and Materials. Shown are representative autoradiograms for the vehicle-treated Ctl, MS, CIS, and MS + CIS groups, as well as the ketanserin-treated Ctl, MS, CIS, and MS + CIS groups **(B)**. The robust induction of *Arc* messenger RNA (mRNA) levels in the prefrontal cortex observed in the vehicle-treated MS + CIS group was not observed in the MS + CIS group that received postnatal Ket treatment. Postnatal Ket treatment to Ctl animals did not alter *Arc* mRNA levels as compared with vehicle-administered control animals. Results are expressed as percent of the control + vehicle group and are the mean ± SEM (*n* = 5–10 per group). (^$^*p* < .01 significantly different from MS, ^δ^*p* < .05 significantly different from CIS; nonparametric Kruskal-Wallis test and Dunn's post hoc test). P, postnatal day.

We next sought to examine whether postnatal ketanserin treatment was capable of normalizing the dysregulated pattern of prefrontal *Arc* mRNA expression evoked by CIS in MS animals (Figure 4A). MS animals administered vehicle in early life showed a robust and significant induction of *Arc* mRNA levels in the PFC following CIS exposure. In striking contrast, ketanserin treatment prevented the upregulation of *Arc* mRNA observed following CIS administration to MS animals (Figure 4B). The Kruskal-Wallis test revealed that postnatal ketanserin treatment to MS animals significantly prevented the aberrant pattern of CIS-evoked *Arc* mRNA expression in the PFC (Figure 4B). We did not observe any influence of postnatal ketanserin treatment on baseline *Arc* mRNA expression in the control groups.

### Postnatal Ketanserin Treatment Prevents Specific Transcriptional Changes Observed in the Prefrontal Cortex of Maternally Separated Animals

MS animals exhibit a perturbed transcriptome in the PFC in adulthood, including alterations in genes associated with G-protein signaling (protein kinase C: *Prkcb1*, pleckstrin: *Plek*, calcineurin: *Ppp3ca*, phospholipase C: *Plcd4*) and excitatory synapses (neuroligin 1: *Nlgn1*, *N*-methyl-D-aspartate receptor subunit 2D: *Grin2d*) (17). We addressed whether postnatal ketanserin treatment would prevent the altered expression of these specific genes perturbed in the PFC of adult MS animals (Table 1). Ketanserin treatment during MS prevented the induction of *Prkcb1*, *Plek,* and *Ppp3ca* mRNA in the PFC (Table 1). Two-way ANOVA analysis revealed a significant MS × Ket interaction for *Prkcb1* [*F*(1,27) = 9.46, *p* = .005], *Plek* [*F*(1,27) = 11.63, *p* = .002], and *Ppp3ca* [*F*(1,27) = 17.35, *p* = .0003] mRNA levels. In contrast, two-way ANOVA analysis indicated no significant MS × Ket interaction for *Plcd4* [*F*(1,27) = 2.06, *p* = .16], *Nlgn1* [*F*(1,27) = 3.90, *p* = .06], and *Grin2d* [*F*(1,27) = 2.60, *p* = .12]. Postnatal ketanserin treatment in control animals did not alter the baseline adult expression of these genes in the PFC. Taken together, our results indicate that postnatal ketanserin treatment prevents the altered regulation of specific genes known to be perturbed in the adult PFC following MS.

**Figure 5:**
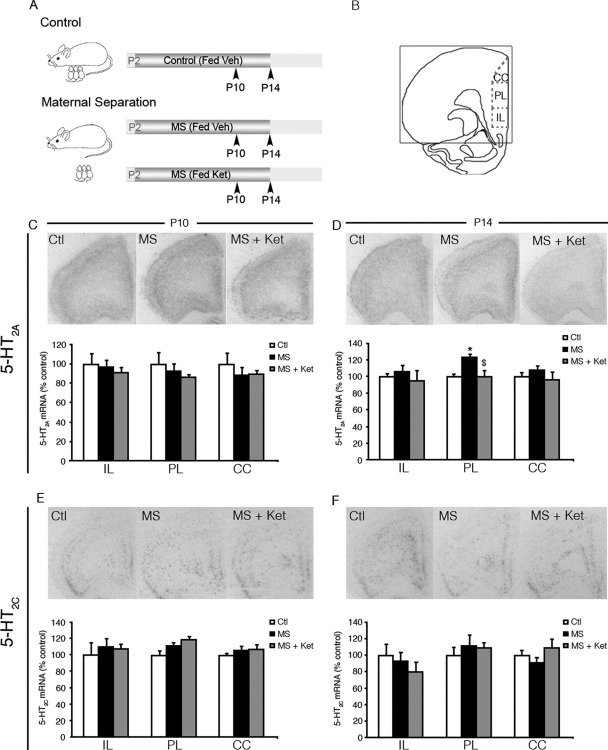
Postnatal ketanserin (Ket) treatment prevents the maternal separation (MS) mediated induction of 5-HT_2A_ messenger RNA (mRNA) at postnatal day 14 (P14) in the prelimbic region of the prefrontal cortex (PFC). Shown is a schematic of the treatment paradigm **(A)**. Pups from vehicle (Veh)-treated control (Ctl), Veh-treated MS, and Ket-treated maternal separation (MS + Ket) groups were assessed for 5-HT_2A_**(C, D)** and 5-HT_2C_**(E, F)** mRNA expression at postnatal day 10 (P10) and P14 in the infralimbic (IL), prelimbic (PL), and cingulate cortex (CC) subdivisions of the prefrontal cortex **(B)** using in situ hybridization. Maternal separation animals exhibited a selective induction in 5-HT_2A_ receptor mRNA levels in the PL subdivision of the PFC at P14 **(D)** and not P10 **(C)**. Interestingly, postnatal Ket treatment during MS prevented this MS-mediated increase in 5-HT_2A_ mRNA levels. 5-HT_2C_ receptor mRNA was found to be unaltered at both P10 and P14 in the PFC of MS animals **(E, F)**. Results are expressed as percent of the Ctl group and are the mean ± SEM (*n* = 3–6 per group). (*p < .05 significantly different from Ctl, ^$^*p* < .05 significantly different from MS + Ket; analysis of variance and Bonferroni post hoc test). 5-HT_2A_, serotonin type 2A; 5-HT_2C_, serotonin type 2C; P2, postnatal day 2.

We next sought to examine whether postnatal treatment with the 5-HT_2_ receptor antagonist ketanserin during MS influences the trajectory of expression of 5-HT_2A_ and 5-HT_2C_ receptors within the PFC (Figure 5A–E). In situ hybridization revealed that 5-HT_2A_ mRNA levels are not regulated by MS at P10 in the IL, PL, or CC divisions of the PFC (Figure 5B). At the end of the MS paradigm on P14, MS animals exhibited significantly enhanced 5-HT_2A_ mRNA levels specifically within the PL subdivision of the PFC (Figure 5C). Interestingly, postnatal ketanserin treatment prevented this MS-associated induction in 5-HT_2A_ mRNA levels [one-way ANOVA: *F*(1,11) = 15.11, *p* = .0007]. In contrast, 5-HT_2C_ receptor expression in the PFC was unaltered in MS animals at both the ages examined (Figure 5D, E).

## Discussion

The early life stress of MS evokes several long-term consequences, including increased adult anxiety behavior (7–9), perturbed stress responses (7,10,11), and transcriptional changes in key limbic neurocircuitry (17). We recently reported that MS animals exhibit enhanced 5-HT_2_ receptor function and increased expression of genes linked to 5-HT_2_ receptor signaling in the PFC (17). The major finding of the present study is that pharmacological blockade of the 5-HT_2_ receptor in postnatal life prevents the emergence of enhanced anxiety in MS animals. Postnatal ketanserin treatment also blocks the enhanced expression of 5-HT_2A_ receptor mRNA observed in the PFC in postnatal life and prevents the upregulation of specific genes linked to 5-HT_2_ receptor signaling (*Prkcb1, Ppp3ca,* and *Plek*) in the PFC of adult MS animals. Further, we show that MS disrupts the pattern of IEG expression evoked by chronic stress in adulthood, and postnatal 5-HT_2_ receptor blockade normalizes this dysregulated pattern. Taken together, our findings demonstrate that postnatal treatment with the 5-HT_2_ receptor antagonist ketanserin prevents the emergence of specific sequelae of MS and implicates 5-HT_2_ receptors in the establishment of affective dysfunction in animals with a history of early stress.

Perturbations of serotonergic neurocircuitry in postnatal life have been strongly linked to the establishment of altered anxiety states in adulthood (13,14,28). Pharmacological studies involving postnatal treatment with serotonin selective reuptake inhibitors result in persistent increases in adult anxiety behavior (29). Conditional genetic loss of function of the forebrain serotonin type 1A (5-HT_1A_) receptor restricted to postnatal life evokes enhanced anxiety in adulthood (30). Further, postnatal pharmacologic blockade of the 5-HT_1A_ receptor is associated with an increase in anxiety (31). In contrast to the anxiogenic state observed in the 5-HT_1A_ receptor knockouts (30) or following postnatal 5-HT_1A_ receptor blockade (31), 5-HT_2A_ receptor knockouts exhibit anxiolytic effects in adulthood (15). However, the consequences of 5-HT_2_ receptor perturbations in postnatal life on the emergence of anxiety behavior in adulthood have not been examined. It is noteworthy that animals with a history of MS exhibit enhanced adult anxiety, accompanied by robust increases in 5-HT_2_ receptor function that emerge soon following MS and persist into adulthood (17). Further, we demonstrate that during postnatal life MS animals exhibit a significant increase in 5-HT_2A_ mRNA levels within the prelimbic subdivision of the PFC. Strikingly, we find that postnatal ketanserin treatment blocks both the MS-evoked changes in prefrontal 5-HT_2A_ mRNA expression in postnatal life, as well as the emergence of anxiety behavior following MS in adulthood. These results raise the possibility of a link between enhanced 5-HT_2_ responses and the establishment of increased anxiety that arises from adverse early experience. However, while interpreting these results, it is important to note that ketanserin, which has a high affinity and selectivity for the 5-HT_2_ receptor over other serotonergic and dopaminergic receptors, does not show a strong selectivity for the 5-HT_2A_ over the 5-HT_2C_ receptor subtype and is reported to have a moderate affinity for the α1 adrenergic receptor (32).

While postnatal ketanserin treatment blocks anxiety behavior in MS animals, it does not influence baseline anxiety in control animals, in striking contrast to studies demonstrating a robust effect on baseline adult anxiety following postnatal 5-HT_1A_ receptor (30) or serotonin transporter blockade (29). A possible explanation for why ketanserin treatment influences anxiety behavior only in MS animals and not in control animals may be because MS is associated with elevated 5-HT_2_ receptor function (17). This suggests that the effects of postnatal ketanserin treatment may predominantly modulate pathological anxiety states that arise as a consequence of early stress. The 5-HT_2_ receptor is a strong candidate for mediating stress-induced alterations in behavioral affect (33–36). 5-HT_2_ receptors are putative targets for stand-alone (37,38) or adjunct antidepressant treatments (39,40). 5-HT_2_ receptor-mediated signaling is enhanced in patients with affective disorders (35) and in animal models of both early life (17) and adult-onset chronic stress (41,42). Interestingly, both early and adult stress evoke increases in prefrontal 5-HT_2A_ receptor expression and function (17,43,44), suggesting that independent of the timing of the stressor, a modulation of 5-HT_2_ receptor function may serve to mediate the effects of stressful life experiences in shaping individual differences in vulnerability for adult psychopathology.

Adverse early life history is also known to evoke dysregulated neuroendocrine responses to adult stress (7,10,11) that have been hypothesized to contribute to the exacerbated risk for stress-related affective dysfunction. However, relatively little is known about the influence of early stress on the pattern of central circuit activation by adult stressors. Using IEG expression to profile the pattern of neuronal circuit activation, adult stressors show robust induction in *Arc* and *c-fos* mRNA in key limbic neurocircuitry, including the PFC (25,45). The PFC plays an important role in integrating the emotional salience of a stressful stimulus with eventual behavioral output (46–49). Strikingly, the pattern of induction of IEGs in the PFC evoked by single stress exposure habituates following repeated experience of the same stress (27). We provide novel evidence that a history of MS results in a failure to exhibit this habituation of *Arc* regulation in the PFC following repeated stress in adulthood. This suggests that chronic stress may continue to be perceived as novel in animals with adverse early life experience or alternatively that mechanisms that regulate the habituation of prefrontal circuits following chronic stress may be dysfunctional in MS animals. Given the top-down control of the PFC in modulating anxiety (50), altered prefrontal circuit activation in MS animals may have a significant effect on stress-evoked anxiety responses. It is interesting to speculate that the perturbed stress sensitivity of prefrontal circuits in MS animals may then contribute to the predisposition for psychopathology, possibly by influencing the prefrontal control of key subcortical circuits including the amygdala (51,52). We find that postnatal 5-HT_2_ receptor blockade normalized the differential pattern of *Arc* expression in prefrontal cortical circuits evoked by chronic stressors in MS animals. Taken together, our results demonstrate the ability of transient postnatal ketanserin treatment to prevent both enhanced anxiety and the perturbed pattern of stress-induced IEG response observed in MS animals. While largely speculative, this opens up the possibility that enhanced 5-HT_2_ receptor function in the PFC may contribute to both the increased anxiety and the perturbed pattern of stress-induced prefrontal circuit activation.

While we have demonstrated that postnatal ketanserin treatment blocks both anxiety and dysfunctional patterns of stress-evoked IEG responses, at this juncture it is difficult to pinpoint specific neuronal circuits that may be targeted by systemic ketanserin treatment to mediate its effects. We addressed whether MS evokes a change in prefrontal expression of 5-HT_2A_ and 5-HT_2C_ receptors and whether MS-induced changes are influenced by postnatal ketanserin treatment. MS animals showed a selective induction of prelimbic 5-HT_2A_ receptor mRNA levels at P14, an effect blocked in MS animals that received postnatal ketanserin treatment. Interestingly, MS animals in adulthood do not show any change in the mRNA levels of either 5-HT_2A_ or 5-HT_2C_ receptors (17), suggesting a relatively transient effect of MS on prefrontal 5-HT_2A_ receptor expression. However, adult MS animals exhibit robustly enhanced 5-HT_2A_ receptor function, likely through enhanced expression of downstream signaling pathways, including enhanced expression of genes such as protein kinase C—*Prkcb1,* calcineurin—*Ppp3ca,* and phospholipase C—*Plcd4* (17). We also find that postnatal ketanserin treatment prevents the enhanced expression of specific genes (*Prkcb1, Ppp3ca*) implicated in altered 5-HT_2A_ receptor signaling (53–55) in the PFC of MS animals in adulthood. Increased protein kinase C function is implicated in the prefrontal dendritic spine loss and working memory deficits associated with chronic stress (56). Ketanserin treatment, by preventing the enhanced expression of calcineurin and *Prkcb1*, may serve to ameliorate the enhanced vulnerability to chronic stress-evoked pathology and the increased 5-HT_2_ receptor function in MS animals. We also observed that ketanserin treatment did not block the disrupted regulation of specific MS-evoked gene expression changes (*Nlgn1*, *Grin2d*, *Plcd4*). One can envisage that the effects of ketanserin on behavioral consequences of MS may arise mechanistically, either through a prevention of underlying molecular events that establish perturbed anxiety or through the induction of adaptive compensatory changes that counteract MS-evoked anxiety-inducing molecular mechanisms.

Previous studies have predominantly focused on reversing in adolescence or adulthood the adverse effects of MS on anxiety behavior using both enriched environment (57) and chronic antidepressant administration (8,58). However, relatively few studies have involved interventions that overlap with the period of MS and may serve to negate the emergence of adverse consequences of early stress exposure. A single report indicates that pharmacologic treatment with a neuroactive steroid, tetrahydrodeoxycorticosterone, during MS can block the behavioral and neuroendocrine sequelae of early stress (59). Given the strong interrelationship between neuroactive steroid pathways and 5-HT_2_ receptors (60–62), it is possible that these systems may play a critical role in postnatal life in mediating the consequences of early stress. Evidence of a reciprocal relationship between 5-HT_2_ receptors and corticotrophin releasing factor (63) raises the possibility that these systems may also cross-sensitize their responses to stress exposure.

In summary, our data clearly demonstrate that postnatal ketanserin treatment prevents the emergence of anxiety following MS, and along with prior results, strongly link perturbed 5-HT_2_ receptor function following MS to the establishment of susceptibility for adult anxiety. Given the increasing need for early treatment interventions that prevent the development of affective vulnerability, our results underscore the importance of 5-HT_2_ receptors as potential therapeutic targets.

## Figures and Tables

(Panel redrawn with permission from Paxinos and Watson [64], copyright Elsevier 1997.)

**Table 1:** Postnatal Treatment with Ketanserin Prevents Specific Transcriptional Changes Induced by Maternal Separation in the Adult Prefrontal Cortex

Gene	Ctl	MS	Ket	MS + Ket
*Prkcb1*	1.00 ± .02	1.22 ± .04^a^	1.04 ± .10	1.01 ± .13^b^
*Plek*	1.00 ± .07	1.25 ± .03^a^	1.12 ± .08	1.03 ± .13^b^
*Ppp3ca*	1.00 ± .04	1.17 ± .03^a^	1.06 ± .02	.99 ± .04^b^
*Plcd4*	1.00 ± .03	1.24 ± .05^a^	.99 ± .13	1.07 ± .19
*Nlgn1*	1.00 ± .09	1.30 ± .05^a^	1.20 ± .09	1.22 ± .16
*Grin2d*	1.00 ± .05	1.28 ± .03^a^	1.15 ± .15	1.29 ± .18^a^

Animals from the control (Ctl) and maternal separation (MS) groups received treatment from postnatal days 2 to 14 with either the serotonin type 2 receptor antagonist ketanserin (Ket) or vehicle, and gene expression was examined at postnatal day 90. We examined the expression of genes of specific interest implicated in G-protein signaling and known to contribute to serotonin type 2 receptor-mediated signal transduction, cellular excitability, and neuronal plasticity. Quantitative polymerase chain reaction analysis for the following genes, protein kinase C (*Prkcb1*), pleckstrin (*Plek*), calcineurin (*Ppp3ca*), phospholipase C (*Plcd4*), neuroligin 1(*Nlgn1*), and *N*-methyl-D aspartate-receptor subunit 2D (*Grin2d*) was performed from the prefrontal cortex as described in Methods and Materials. The expression of these transcripts was significantly induced in the prefrontal cortex of MS animals in adulthood. Postnatal Ket treatment blocked the messenger RNA induction of *Prkcb1*, *Ppp3ca,* and *Plek* but not of *Plcd4*, *Nlgn1,* and *Grin2d* in the prefrontal cortex. Data are expressed as fold change and are the mean ± SEM (*n* = 5–10 per group). (Analysis of variance and Bonferroni post hoc test).

a *p* < .05 compared with Ctl.

b *p* < .01 significantly different from MS.
