# Supplementary material for: Postnatal Serotonin Type 2 Receptor Blockade Prevents the Emergence of Anxiety Behavior, Dysregulated Stress-Induced Immediate Early Gene Responses, and Specific Transcriptional Changes that Arise Following Early Life Stress
Source: Biol Psychiatry. 2011 Dec 1;70(11):1024–32. doi: 10.1016/j.biopsych.2011.08.005 (PMC3210326; doi:10.1016/j.biopsych.2011.08.005)
Supplement: Supplement 1 [file mmc1.pdf]

Postnatal 5-HT_2 receptor blockade prevents the emergence of anxiety behavior, dysregulated stress-induced immediate early gene responses and specific transcriptional changes that arise following early life stress

Supplemental Information

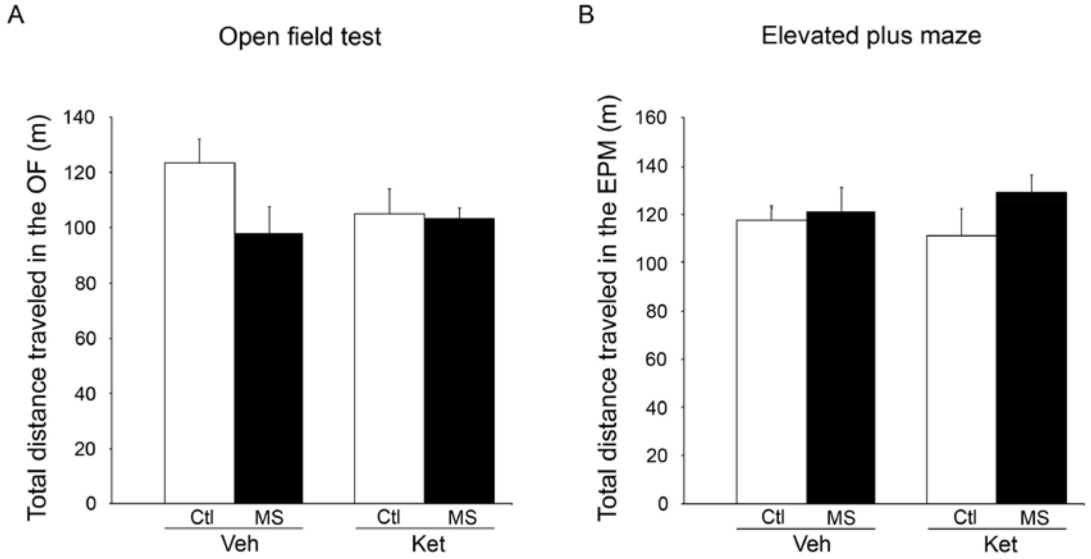

Figure S1. Postnatal treatment with the 5-HT_2 receptor antagonist ketanserin (Ket) in either control or maternal separation groups does not alter (**A**) the total distance traveled in the open field (OF) or (**B**) in the elevated plus maze (EPM) test. Shown are graphical representations of the total distance traversed by control (Ctl) and maternal separation (MS) groups administered vehicle (Veh) or Ket in early postnatal life when tested for anxiety behavior in adulthood on the open field test over a duration of 15 minutes and in the elevated plus maze over a duration of 30 minutes. The results are expressed as the mean ± SEM total distance traveled in the open field or the elevated plus maze (n = 4-7/group).

Table S1. List of TaqMan assays used for quantitative polymerase chain reaction (qPCR). Shown in the table is the list of TaqMan assays used for qPCR validation of microarray results.

TaqMan Assay ID	Gene Symbol	Gene Name
Hs99999901_s1	*18S*	18 s RNA
Rn00667869_m1	*Actb*	beta actin
Rn99999916_s1	*Gapdh*	glyceraldehyde-3-phosphate dehydrogenase
Rn00575638_m1	*Grin2d*	glutamate receptor, ionotropic, *N*-methyl D-aspartate 2D
Rn01527840_m1	*Hprt1*	hypoxanthine-guanine phosphoribosyl transferase 1
Rn00587892_m1	*Nlgn1*	neuroligin 1
Rn00589857_m1	*Plcd4*	phospholipase C, delta 4
Rn01429661_m1	*Plek*	pleckstrin
Rn00566855_m1	*Ppp3ca*	protein phosphatase 3, catalytic subunit, alpha isoform
Rn00562312_m1	*Prkcb1*	protein kinase C, beta 1
